# Infective endocarditis caused by *Paenibacillus thiaminolyticus*: a case report and review of literature

**DOI:** 10.1093/ehjcr/ytad566

**Published:** 2023-11-17

**Authors:** Filip Depta, Martin Pažitný, Michal Trebišovský, Tatiana Maďarová, Jana Deptová

**Affiliations:** Department of Critical Care, East Slovak Institute for Cardiovascular Diseases, Ondavská 8, Košice 040 11, Slovakia; Medical Faculty, Pavol Jozef Šafárik University, Trieda SNP 1, Košice 040 11, Slovakia; Department of Critical Care, East Slovak Institute for Cardiovascular Diseases, Ondavská 8, Košice 040 11, Slovakia; Medical Faculty, Pavol Jozef Šafárik University, Trieda SNP 1, Košice 040 11, Slovakia; Medical Faculty, Pavol Jozef Šafárik University, Trieda SNP 1, Košice 040 11, Slovakia; Department of Cardiac Surgery, East Slovak Institute for Cardiovascular Diseases, Košice, Slovakia; Department of Cardiac Surgery, East Slovak Institute for Cardiovascular Diseases, Košice, Slovakia; St.Elizabeth College of Health and Social Work, Námestia 1. mája 1, Bratislava 811 06, Slovakia; Medical Faculty, Pavol Jozef Šafárik University, Trieda SNP 1, Košice 040 11, Slovakia; Department of Internal Medicine, Louis Pasteur University Hospital, Košice, Slovakia

**Keywords:** *Paenibacillus thiaminolyticus*, Infective endocarditis, Mitral valve, Cardiac surgery, Case report

## Abstract

**Background:**

*Paenibacillus* constitutes a genus of gram-positive, facultatively anaerobic, rod-shaped bacteria that act as potentially opportunistic pathogens. With only a few documented case studies to date, *Paenibacillus* species are rarely the cause of a disease in humans.

**Case summary:**

We report a case involving a 64-year-old male with known mild mitral regurgitation, who presented with fever and dyspnoea. Initially treated with empirical antibiotics, his blood cultures cultivated *Paenibacillus thiaminolyticus*, a previously unreported cause of endocarditis. Transoesophageal echocardiography demonstrated vegetations on the both leaflets of mitral valve along with severe mitral regurgitation, thus confirming a diagnosis of endocarditis. The patient was referred for cardiac surgery; however, the procedure was delayed due to complications related to a known hepatic cyst and additionally contraction of COVID-19 infection. The patient subsequently underwent mitral valve replacement without complications.

**Discussion:**

Because of its rarity, guidelines to recommend specific antibiotics to treat *Paenibacillus* infective endocarditis are absent. To confirm the pathogen, molecular methods such as mass spectrometry or 16S rRNA sequencing are required. Early targeted antibiotic therapy and cardiac surgery are warranted to achieve good clinical outcomes.

**Learning points**

- *Paenibacillus thiaminolyticus* is a rare, yet possible, cause of endocarditis in humans.
- Definitive diagnosis relies upon advanced molecular methods, such as mass spectrometry or 16S rRNA gene sequencing.
- Although sensitivity to antimicrobial drugs varies, strains usually show high resistance to vancomycin.

## Introduction

*Paenibacillus* species represent a Gram-positive, rod-shaped, facultative anaerobic bacteria that are rare causes of disease in humans. *Paenibacillus thiaminolyticus* was only discovered in 2008, and case reports attributing aetiology to this infectious agent are extremely rare.^1^ To the best of our knowledge, endocarditis of the native heart valve caused by *P. thiaminolyticus* has not been described in the literature to date.

## Summary figure

**Table:** 

**4 months prior**	Routine transthoracic echocardiogram (TTE) checkup for known mild mitral regurgitation without progression compared with the previous examinations
**Initial presentation (Day 0)**	Admission due to 4-day history of fever and mild dyspnoea at rest
**Day 3**	Confirmation of *Paenibacillus thiaminolyticus* with mass spectrometry from the bloodstream samples
**Day 4**	Transoesophageal echocardiography revealing severe mitral regurgitation with vegetations on both leaflets of the mitral valve Computed tomography of the abdomen revealing suspected bleeding into a large hepatic cyst
**Day 10**	Laparoscopic surgery with hepatic cyst resection
**Day 32**	Mild COVID-19 symptoms
**Day 130**	Cardiac surgery (mitral valve replacement with bioprosthesis)
**Day 140**	Discharged from hospital

## Case report

In August 2022, a 64-year-old obese [body mass index (BMI) 38] male was referred to the emergency department with a 4-day history of fever (38.2°C) and mild dyspnoea at rest. His heart rate was regular, with sinus rhythm at 99 beats/min, blood pressure (BP) of 122/79 mmHg, and SpO_2_ of 90% while breathing room air. Auscultation of the heart revealed holosystolic murmur (4/6), which was loudest at the heart apex. His past medical history included arterial hypertension and known mild mitral regurgitation [effective regurgitant orifice area (EROA) < 0.2 cm^2^] with preserved ejection fraction of the left ventricle. He also had multiple hepatic cysts (three smaller and one large, measuring 20 × 17 cm) and was scheduled for elective surgery.

Initial laboratory markers of sepsis found elevated C-reactive protein (CRP) (223 mg/L), procalcitonin (0.53 μg/L), fibrinogen (6.3 g/L), and normal white blood cell count of 7.9 × 10^9^/L. He was also found to have severe sideropenic anaemia with haemoglobin of 75 g/L and haematocrit of 0.26 at admission. All other routinely measured laboratory parameters were within normal range.

Because of high probability of bacterial infection, blood cultures were obtained, and the patient was started on empirical intravenous antimicrobial drug therapy consisting of a combination of amoxicillin/clavulanate and gentamycin, according to the local hospital protocol. Despite these treatments, fever persisted, and the CRP level increased to 267 mg/L over the next 3 days.

Blood cultures were obtained for microbiology diagnostics using BACTEC (BD BACTEC Plus Aerobic and BD BACTEC Plus Anaerobic) cultivation media. Both aerobic and anaerobic bottles were cultured at 37°C on blood agar and Schaedler agar for 72 h. The anaerobic bottle showed colonies of viable gram-positive rod-shaped bacteria. Subsequently, matrix-assisted laser desorption/ionization time-of-flight mass spectrometry (MALDI-TOF MS, Bruker Daltonics, Wissembourg, France) analysis was performed and the pathogen was identified as *P. thiaminolyticus*. All other cultivations from skin, nose, throat, rectal swabs, as well as urine samples returned either normal flora or were negative. Susceptibility to common antimicrobial agents was determined via an automated antimicrobial susceptibility testing system (VITEK system, bioMérieux, France). After obtaining susceptibility results, antibiotic therapy was switched from amoxicillin/clavulanate to clindamycin for 6 weeks (Supplementary material S1).

**Figure 1:**
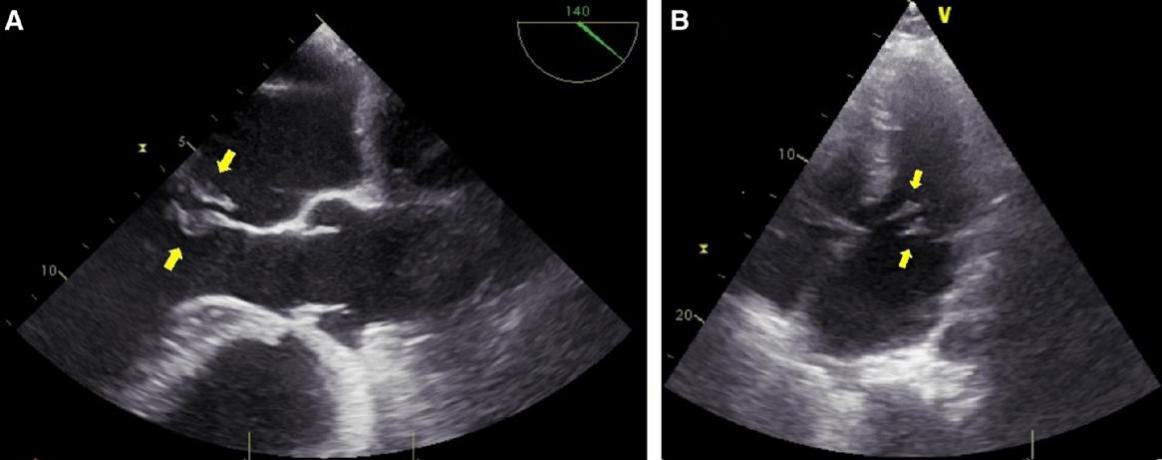
(*A* and *B*) Transoesophageal echocardiography mid-oesophageal aortic valve long-axis (ME AV LAX) and transthoracic echocardiogram apical 5C view at the time of admission showing vegetations on both leaflets of the mitral valve (arrows), with largest vegetation having the size of 8 × 3 mm.

**Figure 2:**
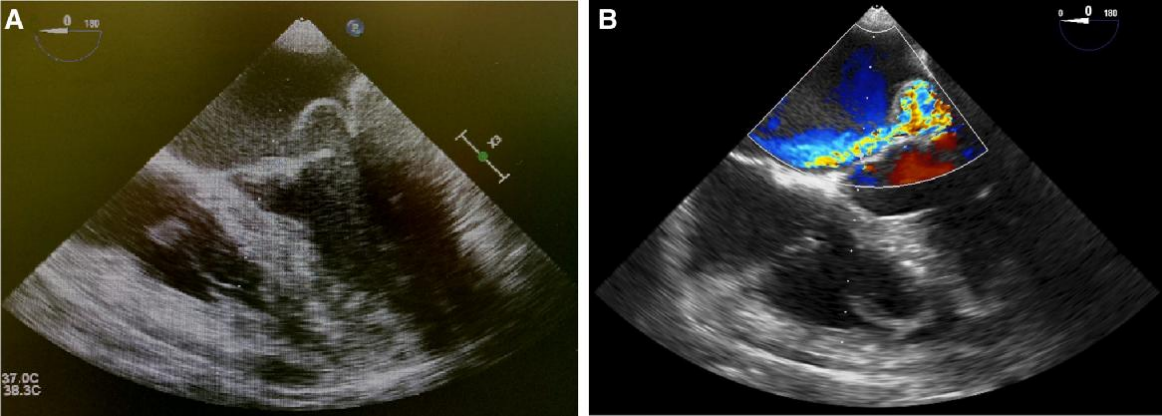
(*A* and *B*) Peri-operative transoesophageal echocardiography mid-oesophageal four-chamber (ME 4C) view showing severe eccentric mitral regurgitation with prolapsed P2 scallop causing Coanda effect.

Due to the holosystolic murmur and positive blood cultures, transoesophageal echocardiography (TOE) was performed and revealed numerous vegetations on both leaflets of the mitral valve (largest vegetations measuring 8 × 3 mm), thus confirming infective endocarditis (IE) (*Figure 1A* and *B*; Supplementary material S2). Further assessment of the mitral valve showed severe eccentric mitral regurgitation (EROA 0.6 cm^2^) with prolapsed P2 scallop causing Coanda effect (Carpentier classification type II) (*Figure 2A* and *B*). The size of the mitral ring was 3.9 × 4.1 cm. Other heart valves were not affected by IE. The patient was therefore scheduled for cardiac surgery to replace the mitral valve.

However, because of the history of large hepatic cysts and severe anaemia at admission, a computed tomography scan of the abdomen was performed and showed suspected bleeding into the largest hepatic cyst (*Figure 2*). Bleeding was taken as a higher priority over the cardiac surgery. While covered with antibiotics for IE, the patient successfully underwent laparoscopic surgery with cyst resection. Cultivations from the hepatic cysts returned negative results for bacteria.

**Figure 3:**
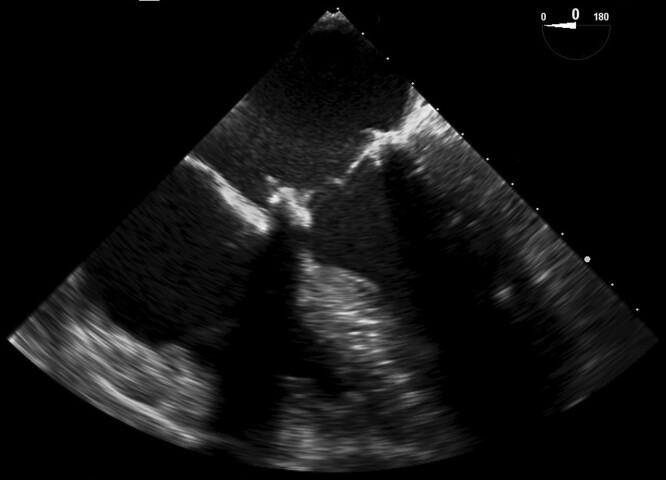
Peri-operative transoesophageal echocardiography mid-oesophageal four-chamber (ME 4C) view showing replaced mitral valve with bioprosthesis.

In January 2023, 5 months after the diagnosis of IE, he ultimately underwent mitral valve replacement. Surgery was initially delayed due to recovery from hepatic cyst removal and later also due to contracting COVID-19, from which he recovered completely. Peri-operative inspection showed post-inflammatory changes of both leaflets of the mitral valve, dilated mitral anulus, and ruptured tendinous cords of the P2 and P3 scallops. Consequently, after proper sizing, a mitral bioprosthesis (Medtronic Mosaic, N.31) was placed in the mitral position (*Figure 3*).

The infected mitral valve was sent to microbiology for cultivation, but 5 months after the diagnosis had been established, the results came back negative for any bacteria. The surgery as well as the rest of the post-operative course was uneventful, and he was released from the hospital in good condition on the 10th post-operative day. On routine post-operative follow-up 6 months after discharge from the hospital, the patient appears well and is able to resume routine daily activities without limitations.

## Discussion

Infective endocarditis is an infection of the endocardial surface of the heart, usually affecting one or more cardiac valves, leading to severe valvular insufficiency, congestive heart failure, and/or myocardial abscesses. Treatment involves a long course of antibiotics (usually 6 weeks) and often requires cardiac surgery to replace the damaged valves. Our case report describes *P. thiaminolyticus* as a rare cause of IE. Two other cases of the *Paenibacillus* group have been recently reported to be confirmed cause of IE. These are *Paenibacillus provencensis* affecting the native mitral valve, *Paenibacillus glucanolyticus* causing blood stream infection in a patient with a cardiac pacemaker, and *Paenibacillus popilliae* responsible for IE of aortic valve causing complete heart block (*Table 1*).^2–4^

**Table 1:** Documented cases of infective endocarditis caused by *Paenibacillus* species

Author	Species	Age	Sex	Pathology	Antibiotics	Diagnosis	Outcome
Ferrand *et al*.^2^	*P. glucanolyticus*	65	F	Pacemaker pocket infection	Ceftriaxone	MALDI-TOF MS and 16S rRNA gene sequencing	Recovery
Pinho-Gomes *et al*.^3^	*P. provencensis*	70	F	Mitral valve endocarditis	Vancomycin, Meropenem, Daptomycin	16S rRNA gene sequencing	Recovery
Wu *et al*.^4^	*P. popilliae*	57	M	Aortic valve endocarditis	Penicillin G, Gentamycin	Routine microbiology	Recovery

MALDI-TOF, matrix-assisted laser desorption/ionization time-of-flight mass spectrometry; IE, infective endocarditis.

*Paenibacillus* species represent common opportunistic bacteria that rarely cause disease in humans. These bacteria have been found and cultivated in various places such as soil, fresh and salt water, humus, compost, food, and plants.^5^ In 2008, *P. thiaminolyticus* was first discovered in a patient on haemodialysis with an infected permcath, where bacteria were identified in blood cultures using biochemical and genetic testing.^1^ Since then, *P. thiaminolyticus* have been isolated from blood, surgical site infection, and cerebrospinal fluid (*Table 2*).^1,6–9^

**Table 2:** Cases of human infection caused by *Paenibacillus thiaminolyticus*

Author	Published (years)	Age	Sex	Pathology	Antibiotics	Pathogen confirmation	Outcome
Ouyang *et al*.^1^	2008	80	M	Blood stream infection	Piperacillin/tazobactam and amikacin	16S rRNA gene sequencing	Recovery
Hunt *et al*.^6^	2021	25 days		Meningitis	N/A	N/A	Death
Di Micco *et al*.^7^	2021	33	F	Wound infection	Amoxicillin/clavulanate	MALDI-TOF MS	Recovery
Ericson *et al*.^8^	2022	33 neonates		Neonatal sepsis	Variable	PCR	Variable
Smallcomb *et al*.^9^	2022	16 d	M	Meningo-encephalitis	Ampicillin, ceftazidime, meropenem	MALDI-TOF MS	Recovery

MALDI-TOF, matrix-assisted laser desorption/ionization time-of-flight mass spectrometry; IE, infective endocarditis; PCR, polymerase chain reaction.

The precise identification of rare bacteria poses a challenge for clinicians. Due to its rarity, this opportunistic human pathogen is not widely known to clinicians. Moreover, because of its resemblance to other gram-positive bacteria, it needs to be confirmed by molecular methods that are frequently used in clinical microbiology, such as MALDI-TOF MS. It has been shown that these molecular methods operate with comparable precision at identifying *Paenibacillus* species compared to extended phenotypic methods.^10^ When available, 16S rRNA gene sequencing should be used to confirm the pathogen. In our case, routine cultivations from the mitral valve leaflets returned negative, because the time between initial confirmation of IE and surgery, where samples were obtained, was very long (i.e. 130 days). Moreover, the long initial course of antibiotic therapy was also likely responsible for negative cultivations at the time of surgery.

Various susceptibilities to antibiotics exist across published cases to treat *P. thiaminolyticus*. Guidelines for IE caused by rare bacteria that appear on case-by-case basis do not usually provide guidance for choosing antimicrobial agents. Therefore, it is difficult to recommend specific antibiotics as well as treatment duration. In our case, the bacteria showed good sensitivities to ciprofloxacin, clindamycin, and meropenem but resistance to vancomycin. Although the resistance of *P. thiaminolyticus* to vancomycin has also been reported previously, this seems not to be true for other Paenibacilli.^2,3^*Paenibacillus thiaminolyticus* in our case was suspected to be resistant to amoxicillin/clavulanate as after its empirical use, the patient’s fever did not resolve and CRP levels continued to rise from 223 to 267 mg/L over the next 3 days.

## Conclusions

In summary, our case demonstrates that *P. thiaminolyticus* is a potential cause of IE in humans. Confirmation of pathogens includes advanced methods such as MALDI-TOF mass spectrometry and 16S rRNA gene sequencing. Although various resistances have been reported across the literature, potent penicillin antibiotics or clindamycin seems to be reasonable first-choice agents.

## Supplementary Material

ytad566_Supplementary_Data

## Data Availability

The data underlying this article will be shared upon reasonable request to the corresponding author.

## Lead author biography

Dr Filip Depta is consultant intensivist at the East Slovak Institute for Cardiovascular Diseases. His main interests are cardiac anaesthesia and intensive care haemodynamics with main research focus on cardiopulmonary interactions in mechanically ventilated patients.
