# Supplementary material for: Infective endocarditis caused by Paenibacillus thiaminolyticus: a case report and review of literature
Source: Eur Heart J Case Rep. 2023 Nov 17;7(11):ytad566. doi: 10.1093/ehjcr/ytad566 (PMC10686528; doi:10.1093/ehjcr/ytad566)
Supplement: ytad566_Supplementary_Data [file ytad566_supplementary_data.zip › Supplementary material S1.docx]

**Supplement Table S1**: Sensitivity of antimicrobial drugs for Paenibacillus thiaminolyticus.

| Antibiotic | MIC (mg/L) ^a^ | Sensitivity/Resistance |
| --- | --- | --- |
| Ciprofloxacin | 0.125 | Sensitive |
| Clindamycin | 0.38 | Sensitive |
| Meropenem | 0.38 | Sensitive |
| Vancomycin | 12 | Resistant |

**Abbreviations**: ^a^ MIC – minimum inhibitory concentration
